# Meaningfulness, feasibility, and usability of quality-of-care measures for maternal and infant health: A structured mixed-methods review

**DOI:** 10.1017/cts.2024.681

**Published:** 2024-12-18

**Authors:** Ryan P. Theis, Rahma S. Mkuu, Hannah Marmol, Lauren Silva, Callie Reeder, Jessica Bahorski, Erica Smith, John C. Smulian, Tony S. Wen, Amanda Redinger, Tabresha Blake, Elizabeth A. Shenkman, Dominick J. Lemas

**Affiliations:** 1 Department of Health Outcomes and Biomedical Informatics, College of Medicine, University of Florida, Gainesville, FL, USA; 2 Clinical and Translational Science Institute Learning Health System Program, College of Medicine, University of Florida, Gainesville, FL, USA; 3 Department of Obstetrics & Gynecology, College of Medicine, University of Florida, Gainesville, FL, USA; 4 College of Nursing, Florida State University, Tallahassee, FL, USA; 5 Center for Research in Perinatal Outcomes, University of Florida, Gainesville, FL, USA

**Keywords:** Maternal and infant health, quality measurement, mixed-methods, stakeholder engagement, quality frameworks

## Abstract

**Objectives:**

Improving access to and quality of maternal and infant healthcare are important leverage points to address worsening maternal and infant health disparities in the USA. This study evaluates the comprehensiveness of existing maternal and infant quality-of-care measures to identify aspects of quality that need greater attention in quality measurement.

**Study design:**

We conducted a structured, team-based qualitative review of 88 maternal and infant health measures indexed by the National Quality Forum (NQF), the Agency for Healthcare Research and Quality (AHRQ), the Centers for Medicare and Medicaid Services (CMS), and the National Committee for Quality Assurance (NCQA). We assessed discrete elements relevant to meaningfulness, feasibility, and usability following AHRQ National Quality Strategy (NQS) criteria, with input from researcher, clinician, and citizen scientist investigators. Descriptive statistics on coded measures were calculated using SPSS.

**Results:**

The most common AHRQ NQS priorities addressed were mortality (60%) and safety (48%). Average scores across elements were 59% for feasibility, 61% for practice usability, and 31% for policy usability. Fewer measures addressed coordination, affordability, or patient engagement in the postpartum period. Only 23% of measures were endorsed by NQF, only 17% of measures had publicly available benchmarks, and only 14% had specifications updated in the year prior to review.

**Conclusions:**

Findings from this study can inform the specification of a comprehensive, updated system for maternal and infant quality-of-care evaluation and can facilitate the development of new quality-of-care measures that address underrepresented maternal and infant health issues.

## Introduction

Perinatal health in the USA has been a topic of great concern in recent years [1]. The USA reports higher maternal and infant mortality rates compared to similarly developed countries [2] despite spending substantially more on healthcare [3]. Data on maternal deaths between 2017 and 2019 from 36 US states demonstrate that over 80% of maternal deaths are preventable [4] and could be avoided through improving the quality of maternal care. For example, one-fifth of pregnancy-related mortality associated with hypertensive disorders could be prevented by providing preventive preeclampsia care during the prenatal period [5]. Furthermore, improving quality and safety of care has been shown to substantially improve perinatal morbidity [6].

Access to health insurance is also a significant predictor of morbidity and mortality [7]. Trends in US maternal mortality indicate most maternal deaths occur during the first-year postpartum [2], emphasizing the need to expand Medicaid coverage from 60 days postpartum to up to 12 months postpartum to support delivery of care during the fourth trimester (defined as the first 3 months after birth) [8]. As of March 23, 2023, 30 states, including DC, have expanded coverage to 12 months postpartum and 8 states plan to implement the extension [9]. Although insurance coverage is essential to accessing care, it does not ensure access to high-quality perinatal care. Access to and quality of maternal and infant healthcare are therefore important leverage points for initiatives that address increased incidence and worsening disparities [10].

The Institute of Medicine (IOM) defines quality of care as “the degree to which healthcare services for individuals and populations increase the likelihood of desired health outcomes and are consistent with current professional knowledge [11].” A widely accepted model for healthcare quality follows Avedis Donabedian’s framework, which highlights the relevance of structure, process, and outcomes of care [12]. The World Health Organization further describes seven elements of healthcare quality, acknowledging that healthcare should be effective, safe, people-centered, timely, equitable, integrated, and efficient [13]. These constructs help to guide quality improvement efforts, which include the development and use of measures to evaluate and monitor quality [14]. Research has increasingly engaged patients as stakeholders in quality improvement, informing the development of educational materials, tools, and policy and planning documents, and enhancing care processes [15]. However, there remains a greater need to directly engage patients in the development and evaluation of quality-of-care measures, particularly for measuring quality of perinatal care.

It is therefore essential to identify whether the present landscape of healthcare quality measures is sufficient to inform interventions for current perinatal challenges. Research is also needed to evaluate the appropriateness of existing measures for monitoring perinatal quality of care and outcomes from a multi-stakeholder perspective, including patients and clinicians. This study used a structured review framework to: (1) identify aspects of maternal and infant healthcare that are not sufficiently covered by existing measures and (2) evaluate whether existing measures are meaningful, feasible, and usable for addressing leading perinatal challenges.

## Methods

We conducted a structured, mixed-methods review of known maternal and infant healthcare quality measures, led by an interdisciplinary team of health services researchers, quality-of-care measurement experts, citizen scientists who were health system patients with personal experience receiving perinatal care, and clinicians specializing in obstetrics/gynecology. The team was assembled to ensure the inclusion of diverse perspectives of stakeholders in maternal and infant health, following principles of stakeholder engagement outlined by the Patient-Centered Outcomes and Research Institute (PCORI) [16]. Team members were affiliated with the University of Florida Clinical and Translational Science Institute (UF CTSI) (RPT, AR, TB, EAS, and DJM), were regular collaborators with CTSI researchers (RSM, JB, JCS, and TSW), or were students and residents associated with CTSI researchers and collaborators (HM, LS, CR, and ES). Team members in all disciplines participated in conceptualization of the study, specification of methods for identifying measures, and reviewing measures.

### Measures identification

Table 1 outlines the sources for healthcare quality measures reviewed in this study. Briefly, we identified measures indexed by the National Quality Forum (NQF), the Agency for Healthcare Research and Quality (AHRQ), the Centers for Medicare and Medicaid Services (CMS), and the National Committee for Quality Assurance (NCQA) [17–20]. Measure identification took place between June and September 2020.

**Table 1.** Healthcare quality measure sources

Indexing Organization	Acronym	Measure Count^a^
National Quality Forum	NQF	62
Agency for Healthcare Research and Quality	AHRQ	25
Centers for Medicare and Medicaid Services	CMS	17
National Committee for Quality Assurance	NCQA	13
Measure Steward	Acronym	Measure Count^a^
Agency for Healthcare Research and Quality	AHRQ	5
American Academy of Neurology	AAN	1
American College of Emergency Physicians	ACEP	3
California Department of Public Health	CDPH	1
California Maternal Quality Care Collaborative	CMQCC	2
Center for Patient Safety and Quality Research	CPSQR	1
Centers for Disease Control and Prevention	CDC	7
Centers for Medicare and Medicaid Services	CMS	1
Child and Adolescent Health Measurement Initiative	CAHMI	1
Child Health Corporation of America	CHCA	2
Children’s Hospital of Philadelphia	CHOP	4
Christiana Care Health System	CCHS	1
Collaboration for Advancing Pediatric Quality Measures	CAPQuaM	11
Health Resources and Services Administration	HRSA	1
Hospital Corporation of America	HCA	1
Ingenix	–	3
Leapfrog Group	–	1
Massachusetts General Hospital	MGH	2
National Committee for Quality Assurance	NCQA	13
Pediatric Measure Center of Excellence	PMCoE	7
Physician Consortium for Performance Improvement	PCPI	4
Resolution Health, Inc.	–	1
The Joint Commission	TJC	5
Vermont Oxford Network	VON	6
US Office of Population Affairs	OPA	4

a Measure counts for indexing organization exceed total in study (88) because several measures were listed by more than one organization.

All team members offered input on search terms and search criteria to identify measures. At each resource, we searched for measures using the following terms: “antepartum,” “birth,” “delivery,” “deliveries,” “infant,” “maternal,” “maternity,” “mother,” “neonatal,” “neonate,” “newborn,” “perinatal,” “postpartum,” “pregnancy,” “pregnant,” “prenatal,” and “prepartum.” Both hyphenated (e.g., “post-partum”) and non-hyphenated (e.g., “postpartum”) versions were included in the search.

The search identified 153 unique measures. Measure names and descriptions were reviewed for face validity. Any measure that did not have at least one of the study search terms in the measure name was excluded if it: (1) did not include women of childbearing age (15 through 49 years) and/or infants or toddlers (up through age 3 years); (2) did not address any maternal or infant morbidity/mortality causes or outcomes, or (3) was a simple cost or utilization measure not otherwise associated with a standard of care, which alone cannot be used to evaluate healthcare quality [21].

After exclusions, 88 measures relevant to maternal or infant health were included in the study for further review.

### Measures review

We conducted a concurrent, independent review of published specifications for each measure. These included but were not limited to NQF Quality Positioning System (NQF-QPS) entries, Healthcare Effectiveness Data and Information Set (HEDIS) manuals, and AHRQ Pediatric Quality Measures Program (PQMP) specifications and reports. The review method followed a team-based coding approach and framework analysis methods that are suited for structured qualitative data reduction [22,23]. The framework included elements in five domains:

1. Measure identifiers and specifications included information on the measure steward, date of last update, and the measure numerator and denominator specifications. Additionally, each measure was classified as relevant to the structure, process, or outcomes of care [12]. Each measure was also assigned a focus (women, neonates, infants, and/or toddlers) and a phase (preconception, prenatal, intrapartum, postpartum, and/or interpregnancy).
2. Evidence and support included information on NQF endorsement and ratings and recommendations on clinical practices from the American Academy of Pediatrics (AAP), American College of Obstetricians and Gynecologists (ACOG), Centers for Disease Control and Prevention (CDC), Society for Maternal-Fetal Medicine (SMFM), and US Preventive Services Task Force (USPSTF).
3. Meaningfulness was assessed following the AHRQ National Quality Strategy (NQS) priorities of safety, engagement, coordination, mortality, community, and affordability [24]. Additionally, clinical investigators with subject matter expertise (LS, CR, JB, ES, and TSW) and citizen scientist investigators (AR and TB) rated each measure on its “importance to maternal health” on a 5-point Likert scale.
4. Feasibility was assessed using AHRQ guidance for evaluating measure feasibility, including consistent measure construction and assessment, feasibility of calculating (based on the measure data source and availability of measure diagnosis and procedure codes), and addressing confidentiality concerns [25].
5. Usability was coded in two sub-domains – practice usability (the extent to which providers, clinics, and health systems can incorporate the measure into practice) and policy usability (the extent to which policymakers can use measure findings to inform policy).
  - Practice usability was assessed using AHRQ guidance for evaluating measure usability, including measure presentation, history of use, and compelling content for stakeholder decision-making [25], as well as the availability of measure benchmarks and the level(s) at which the measure is aligned (e.g., provider, facility, and system).
  - Policy usability included the NQS “levers” of feedback, public reporting, learning, certification, consumer incentives, payment, health information technology, innovation, and workforce development [26]. The public reporting element, which specifies whether a measure can be used to inform patient decision-making by comparing performance of providers and clinics, was coded by citizen scientist investigators (AR and TB).

Together, review of these domains and elements allowed us to: (1) identify aspects of maternal and infant healthcare that are not sufficiently covered by existing measures (measure identifiers and specifications, evidence and support); and (2) evaluate whether existing measures are meaningful, feasible, and usable for addressing leading perinatal challenges (meaningfulness, feasibility, and usability).

Measure coding was deductive and followed an iterative cycle for codebook development. The coding team was comprised of two independent reviewers who abstracted information on measure specifications and determined whether measures met the criteria for meaningfulness, feasibility, and usability (RPT and RSM), using a measures framework table developed in MS Excel. Differences in coding were reconciled by consensus between the coders and during team meetings, which included the study lead (DJL), clinical investigators (JCS and TSW), and citizen scientist investigators (AR and TB). A coding lead (RPT) compiled abstracted measure information and updated the final measures framework table during the review period.

Detailed protocols for measure identification and measure coding – including a table listing all reviewed measures – are outlined in **Supplementary Information**.

### Analysis

The final dataset was imported into SPSS (v29) for descriptive statistical analysis. Values for coded elements and constructs were treated as categorical (in most cases, as “yes”/”no” responses). Distributions were calculated for all elements, and most were also stratified according to measure type, focus, and phase. We used the chi-square test for independence to test differences across these categories, with p-values less than 0.05 considered statistically significant.

Society and agency recommendations for clinical practices were reviewed by the coding lead (RPT) and a clinician investigator (ES), and each practice was assigned a single rating: (1) clinical practice guideline; (2) graded recommendation (A or B); (3) graded recommendation (C); (4) general recommendation; (5) proximal recommendation; or (6) no recommendation. A proximal rating was assigned in cases where a clinical practice was recommended but not as described in the measure specifications (e.g., using different populations or follow-up periods). Evidence ratings were added to all corresponding process measures in the dataset.

Overall scores were generated for feasibility, practice usability, and policy usability, representing the percentage of elements met in each domain. Three elements in the policy usability domain were excluded from the analysis. Reviewers determined that the learning and innovation elements could not be coded based on measure specifications alone. Furthermore, the payment element was excluded because of a high rate of “unsure” ratings provided by clinician investigators (average 67%, range 9% to 100%).

Measure importance ratings were collapsed into two categories according to reviewer role. Citizen scientist investigators resolved discrepancies in ratings by consensus to reach a final importance rating for each measure. Ratings by clinical investigators were averaged across raters (excluding those who responded “unsure” or “topic not within specialty”) to reach final clinician importance ratings. In alignment with our team-based coding methodology, citizen scientist and clinician measure importance ratings were compared to identify inter-rater differences that could indicate unique dimensions considered by each reviewer group.

## Results

Table 1 shows the percentage of measures that met the criteria for elements in the evidence and support, meaningfulness, feasibility, and usability domains. Specific measures described in this section are identified by their ID numbers, as listed in the **Supplementary Information**.

**Table 2.** Percentage of measures meeting review criteria, by domain

**Evidence**	**Criterion**	**%**
Strong	Practice established in clinical guidelines or graded “A” or “B”	34%
Moderate	Practice graded “C” or given general recommendation	25%
Proximal	Practice recommended, but not as described in specifications	31%
None	No society or agency recommendation for practice	10%
**Support**	**Criterion**	**%**
Endorsed	Measure endorsed by NQF at the time of review	23%
Not endorsed	Measure never endorsed by NQF at the time of review	28%
Withdrawn	Measure endorsement withdrawn by NQF at the time of review	49%
**Meaningfulness**	**Criterion**	**%**
Safety	Measure addresses a clinical practice that involves risk or harm to the mother or infant	48%
Engagement^a^	Measure addresses a clinical practice that requires treatment adherence by the patient and/or shared decision-making between providers and patients	41%
Coordination	Measure addresses a clinical practice for which effectiveness depends on coordination among providers and/or between providers and patients	18%
Mortality	Measure addresses a risk factor or condition considered to be a leading cause of death of the mother or infant	60%
Community	Measure can inform community-level interventions to improve uptake of preventive health practices	27%
Affordability	Measure can inform efforts to make quality care affordable for individuals, families, employers, and governments	8%
**Feasibility**	**Criterion**	**%**
Consistency	Findings are publicly reported on tests of measure reliability and validity	40%
Calculation (Source)	Measure can be calculated using electronic records, including claims, encounters, or electronic health records	44%
Calculation (Codes)	Measure specifications detail specific diagnosis and/or procedure codes	70%
Confidentiality	Measure does *not* address an aspect of health that has high privacy protections	81%
**Practice usability**	**Criterion**	**%**
Presentation	Specifications define the measure numerator, denominator, and exclusions	84%
History	Measure has history of effective use through established measure sets, endorsement, or validity/reliability testing	61%
Guidance	Measure specifications include detailed guidance for interpreting findings for stakeholders	66%
Alignment	Measure is designed for reporting at the provider or facility level	75%
Benchmarks	Measure has benchmarks for specific populations	17%
**Policy usability**	**Criterion**	**%**
Feedback	Measure can be used to provide performance feedback to providers, facilities, systems, or health plans	97%
Reporting	Measure is useful for public reporting of provider or clinic performance	24%
Certification	Measure can facilitate provider adoption or adherence to approaches to meet safety and quality standards	16%
Consumer^a^	Measure addresses a clinical practice that requires treatment adherence by the patient and/or shared decision-making between providers and patients	41%
HIT	Measure is an “e-measure” or addresses clinical practices that can be delivered via telehealth	11%
Workforce	Measure can inform efforts to invest in health professionals or improve provider network adequacy	6%

a The meaningfulness (engagement) and policy usability (consumer) elements shared the same coding definition.

### Measure characteristics

**Figure 1.**
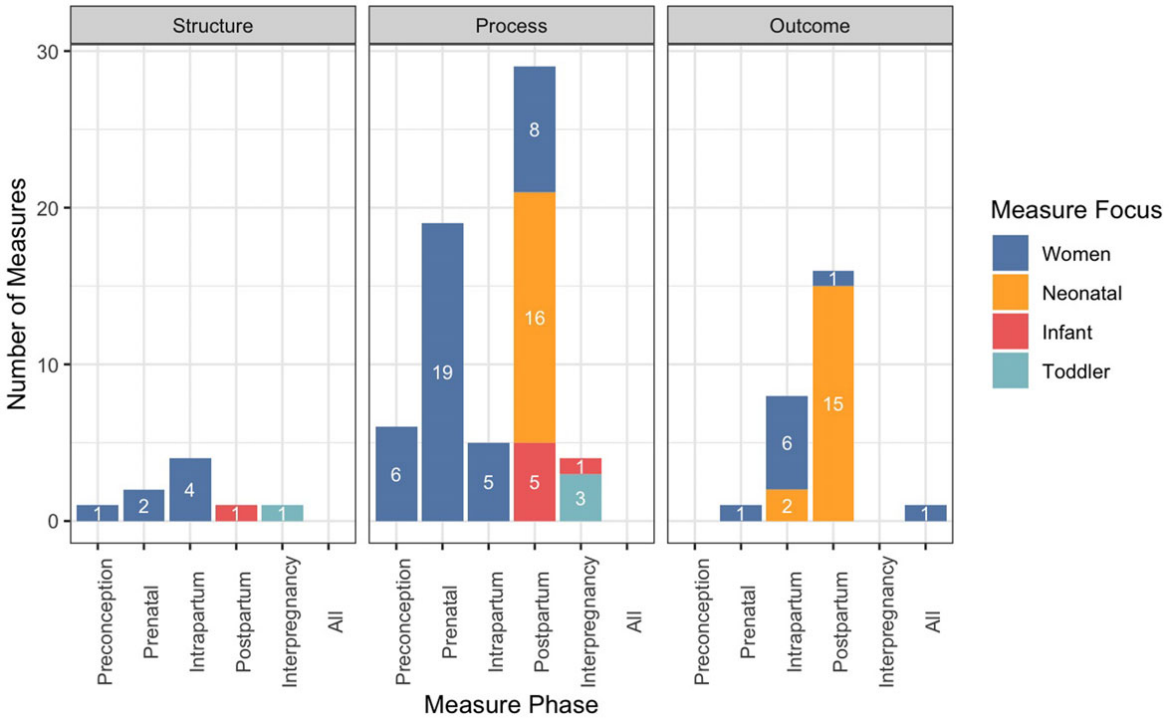
Number of maternal and infant health measures according to quality emphasis, pregnancy phase, and population focus.

Figure 1 provides the distribution of measures according to quality domain, pregnancy phase, and population focus. Most quality measures assessed healthcare processes (64%) or outcomes (27%), while only 9% addressed the structure of care. The most common measure population focus was women (59%), followed by neonates (38%), infants (8%), and toddlers (5%). Nearly half of the measures addressed postpartum care (46%), one-quarter addressed prenatal care (26%), and one-fifth addressed intrapartum care (22%). Measures addressing the preconception (9%) and interpregnancy phases (6%) were less common. While nearly two-thirds of measures had publicly available measure specifications (61%), only 14% had measure specifications that were updated within 1 year before review.

### Evidence and support

Most measures addressed a specific clinical practice (81%). A review of current professional society and government agency recommendations revealed that one-third of these measures (34%) addressed clinical practices that had been formally incorporated into practice guidelines or had received strong evidence ratings (“A” or “B”). One-quarter addressed practices that had received moderate evidence ratings (“C”) or general recommendations (25%). Only 10% of these measures addressed practices for which no society or government recommendations were published. Another one-third of these measures (31%) addressed practices for which society and agency recommendations were proximal, meaning that the practice was recommended but not as described in the measure specifications.

The review also considered NQF endorsement of a measure, as NQF had indexed most measures (70%) at the time of the review. Across all measures, 23% were endorsed by NQF. For nearly half of all measures (49%), NQF had withdrawn endorsement.

### Meaningfulness

The most commonly addressed AHRQ NQS priority was mortality, representing 60% of measures. These included measures that addressed a prevention or treatment practice for any of the top causes of maternal mortality listed for Florida [27] and/or top causes of infant mortality listed for the USA [28]. Examples include measures that address appropriate prophylactic antibiotic use before cesarean section [ID 2], counseling for sudden infant death syndrome [ID 78], and collecting and documenting temperature for low-birth-weight infants [IDs 15, 16, 17]. They also included measures that address risk factors that contribute to the top causes of mortality, such as tobacco use, which is a known risk factor for high blood pressure during pregnancy, increasing the risk of hemorrhage, stroke, and cardiomyopathy.

Safety, defined as “avoiding harm to patients from the care that is intended to help them,” [24] was the second-most common priority, representing nearly half of all measures (48%). For example, two measures addressed prenatal red blood cell antibody testing, which is important for safely dosing Rh immunoglobulin [IDs 66, 67]. Two other measures addressed the incidence of unnecessary episiotomy during delivery, which brings a risk of infection and tearing [IDs 31, 38].

Fewer measures were relevant to maternal or infant care coordination (18%) or affordability (8%). A measure was considered relevant to coordination if it addressed a practice for which effectiveness depends on coordination among healthcare providers or between providers and patients. Examples include measures of the frequency and timeliness of prenatal care [IDs 35, 64.1], availability of certain types of outpatient care for women with high-risk pregnancies [IDs 12, 13], and hearing screening for newborns [IDs 51, 52]. Measures considered relevant to the affordability of maternal and infant healthcare included measures that provide rates of elective deliveries or cesarean births [IDs 53, 54], and one measure of the continuity of newborn insurance coverage [ID 23].

Another element of meaningfulness is the importance of a measure to maternal and infant health. On a 5-point Likert scale, ranging from least to most important, the average importance rating of measures by clinician and citizen scientist investigators was 4.50 (SD = 0.39), ranging from 2.88 to 5.00. There were no significant differences in importance ratings by measure focus or phase. Seven measures received an average rating of 5.00, covering topics related to administration of Rh immunoglobulin in the emergency department [ID 74], immunizations for neonates and toddlers [IDs 20, 46, 50], neonatal intensive care outcomes [ID 49], healthcare-associated bloodstream infections [ID 56], and postpartum depression screening and follow-up [ID 88] (Table 2).

### Feasibility

Fewer than half of the measures in the review had publicly reported findings on validity and reliability tests (40%). The feasibility of calculating measures was determined by assessing whether a measure could be calculated using electronic data, including claims, encounters, and electronic health records (44%), and whether measure specifications included information on diagnosis and/or procedure codes (70%). A significantly lower percentage of postpartum measures could be calculated using electronic data (28%, *p* = 0.004). The percentage of measures relevant to mortality that had published diagnosis and procedure codes was slightly lower (66%). Some measures require data sources that involve higher levels of cost and effort to utilize, including paper records (24%), registries (21%), and surveys (6%).

Measure specifications provide little information on how much a measure may meet confidentiality concerns, as measure reporting is most frequently done at the aggregate level. We instead considered the extent to which a measure may require greater attention to confidentiality based on its use of patient data that has special legal protections, such as data on substance abuse, mental health, or HIV/AIDS. Nearly one in five measures (19%) required the use of data with special protections.

All measures received a score representing the percentage of the four feasibility elements that were met. Feasibility scores ranged from 0% to 100%, with a mean of 59%. Ten measures received a feasibility score of 100%, including measures that address frequency of prenatal care [ID 35], establishment of gestational age [ID 32], cesarean delivery [ID 19], episiotomy [ID 31], complications in newborns (including trauma and bloodstream infections) [IDs 6, 45, 82], low birth weight [ID 42], maternal death rates [ID 29], and immunizations for toddlers [ID 20].

### Practice Usability

Most measures were considered to have effective presentation strategies (84%), based on whether the measure specifications clearly defined the study population, including the measure numerator, denominator, and exclusions.

Nearly, two-thirds of measures were considered to have a history of effective use (61%), including measures that belonged to established measure sets (such as HEDIS or Joint Commission measures), measures that had a history of NQF endorsement, and measures with specifications that demonstrated tests of validity/reliability and use in real-world settings. The percentage of postpartum measures with history of use was significantly higher (75%, *p* = 0.014).

We assessed the extent to which measures had compelling content for decision-making based on three elements. First, 66% of measures had specifications that included detailed guidance for interpreting findings, including evidence from the literature. Second, 75% of measures were designed for reporting at the provider or facility level, which allows findings to inform localized clinical decision-making. The percentage of measures relevant to mortality that could be aligned at the provider or facility level was slightly higher (83%). Third, only 17% of measures had benchmarks for specific populations, which function as standards against which findings can be compared. Among measures relevant to mortality, the percentage that had benchmarks was lower (9%).

All measures received a score representing the percentage of the five practice usability elements that were met. Practice usability scores ranged from 20% to 100%, with a mean of 61%. Six measures received a practice usability score of 100%, including measures that address ultrasound determination of pregnancy location [ID 80], complications in newborns (including trauma and bloodstream infection) [IDs 6, 45], exclusive breastmilk feeding of newborns in the hospital [ID 57], maternal death rates [ID 29], and hearing screening prior to hospital discharge [ID 36].

### Policy usability

Nearly, all measures (97%) are designed in a way to provide performance feedback to providers, facilities, systems, and health plans. Four in 10 measures (41%) have the potential to help consumers adopt healthy behaviors and make informed decisions, based on whether they address practices that require some level of treatment adherence on the part of patients or involve shared decision-making between patients and providers. This element, which corresponds to meaningfulness (engagement), was represented significantly less often in postpartum care measures (25%, *p* = 0.006).

Nearly, one-quarter of measures were considered by citizen scientist investigators to be useful for public reporting of provider or clinician performance (24%). Usefulness for public reporting was significantly higher for measures that address the intrapartum phase (68%, *p* < 0.001) and those that are relevant to safety (35%, *p* = 0.033), and significantly lower for measures that address the postpartum phase (13%, *p* = 0.016).

Fewer measures can be used to certify providers on safety/quality standards (16%) or leverage health information technology as “e-measures” or through addressing clinical practices that can be delivered via telehealth (11%). Very few measures focus on the healthcare workforce (6% overall and 0% of postpartum measures, *p* = 0.036).

All measures received a score representing the percentage of the six policy usability elements that were met. Policy usability scores ranged from 0% to 67%, with an average of 31%. Ten measures received a policy usability score of 67%, including measures that address timeliness of prenatal care [ID 64.1], prenatal immunizations [ID 86], prenatal depression screening and follow-up [ID 87], elective deliveries or cesarean births [IDs 53, 54], exclusive breastmilk feeding of newborns in the hospital [ID 57], postpartum care [ID 64.2], postpartum depression screening and follow-up [ID 88], immunizations for toddlers [ID 20], and anticipatory guidance/family-centered care for mothers of infants and toddlers [ID 70].

### Overall scores

Lastly, we calculated an overall score for each measure based on the combined coding of 20 elements in the meaningfulness, feasibility, practice usability, and policy usability domains. Because the meaningfulness (engagement) and policy usability (consumer) elements shared the same coding definition, policy usability (consumer) was dropped from the calculations to avoid overweighting this dimension. Overall scores could be calculated for 69 measures with valid values for all 20 elements.

Overall scores ranged from 20% to 70%, with an average of 44%. Seven measures received overall scores of 65% or higher, including measures that address the frequency and timeliness of prenatal care [IDs 35, 64.1], cesarean births [ID 54], birth trauma to neonates [ID 6], unexpected complications in newborns [ID 82], postpartum care [ID 64.2], and immunizations for toddlers [ID 20].

## Discussion

Quality-of-care measures are used to assess performance within and across healthcare systems and function as key tools in quality improvement efforts. Using a mixed-methods structured review approach, we identified publicly accessible maternal and infant health quality-of-care measures, coded each measure using a deductive qualitative framework based on constructs in the AHRQ NQS and AHRQ measure evaluation framework, and conducted descriptive statistical analyses of coded constructs across all measures. Together, these methods produced findings that address the study aims of identifying aspects of maternal and infant healthcare that are not sufficiently addressed by existing measures and evaluating the extent to which existing measures are meaningful, feasible, and usable for addressing leading perinatal challenges.

Our study highlights several gaps in the availability of quality indicators that are validated, reliable, and can be linked to assess performance associated with the leading causes of maternal and infant morbidity and mortality. Only one-quarter of measures were endorsed by NQF at the time of the review. This endorsement is based on stakeholder consensus on the extent to which a measure focuses on high-priority areas, can produce reliable and valid results about quality of care, is understandable and relevant to intended users, and uses readily available data sources [29]. Our finding suggests that organizations may be putting significant effort into developing measures that do not meet these standards.

While the majority of measures focus on the process of maternal and infant healthcare, very few measures address the structure of care. In our study, nearly all structure measures were part of the Collaboration for Advancing Pediatric Quality Measures (CAPQuaM) High Risk Obstetrical (HROB) set, which addresses different aspects of preconception, prenatal, and intrapartum care for women. There remains a need for structure measures that focus on infants. A large body of evidence demonstrates that hospital characteristics associated with good quality care, such as staff training, workload, capacity, and neonatologist-to-house staff ratio, are linked to better infant health outcomes [30]. Having a larger set of validated workforce-related measures can help facilities develop and improve capacity, particularly for meeting the needs of women with high-risk pregnancies. Incorporating more measures of structure into quality improvement programs may also bolster gains in patient outcomes that occur in programs focusing on processes of care [31].

Conversely, measures that address outcomes of care largely focused on neonates in the intrapartum and postpartum phases. This finding may be expected, given that a variety of outcomes, including neonatal birth weight, temperatures, and infections, are routinely measured during the period shortly after delivery. The lack of outcome measures focusing on preconception or interpregnancy care may have been a function of our measure identification methods. It is likely that most measures of women’s health outcomes that occur during these periods are not described as maternal care measures and would have been screened out in our selection process. The relatively low number of outcome measures in this study overall aligns with more recent work by SMFM, which found that outcome measures are not always sensitive enough to detect underlying quality issues [32].

With regard to measures of clinical practices, we determined that over half addressed practices that had been incorporated into clinical practice guidelines or given strong to moderate recommendations by national medical societies and organizations. These guidelines and recommendations are based on comprehensive syntheses of evidence that link clinical practices with positive outcomes. However, nearly one-third of these measures did not define clinical practices exactly as outlined in the clinical guidelines and recommendations, and another 10% addressed practices that had no society guidelines or recommendations at all. This finding suggests that a significant number of practice-related measures may not be directly associated with outcomes of care and thus are less likely to provide meaningful information on the impact of quality improvement programs.

Furthermore, few measures addressed the quality improvement priorities of coordination and affordability – priorities that can be effectively addressed using surveys with patients. While surveys can be developed to meet local needs, benchmarking and interpreting measure findings is challenging without validity testing and use in the broader population. Validated surveys, such as the Consumer Assessment of Healthcare Providers and Systems (CAHPS) surveys, include items to collect patient experiences with care coordination. However, existing CAHPS surveys do not address the specific issues encountered by women receiving maternal care [33]. In 2023, AHRQ invited public comment on a potential CAHPS survey to assess patients’ prenatal and childbirth experiences [34]. More recently, the CDC published findings from a study of maternity care experiences using the PN View Moms Survey, focusing on mistreatment, discrimination, and shared decision-making [35]. However, the PN View Moms Survey tool, designed by Porter Novelli Public Services, is proprietary and not available for use by health organizations.

Our study also highlights the quality improvement priorities that are adequately addressed by maternal and infant health measures. Nearly two-thirds of measures are relevant to mortality, nearly one-half address safety of care, and most can be reported at the provider or facility level. Rigorous quality improvement programs, informed by measures in these two priority areas, can help to avert maternal mortality. For example, fewer maternal deaths were observed after implementing protocols, including the formation of an obstetric rapid response team and use of a measure of the severity of obstetric hemorrhage, to improve patient safety at a hospital in New York [36]. Furthermore, nearly half of measures focus on the postpartum period, most of which have an established history of use. This finding aligns with calls for greater emphasis on fourth trimester care [8]. However, there remains a need to improve on the meaningfulness, feasibility, and usability of postpartum care measures, which were less likely to address patient engagement, use electronic records as a data source, or focus on the healthcare workforce.

It is also important to note that measures should be suitable for understanding disparities in quality. Numerous studies report that women from underrepresented racial/ethnic groups are significantly more likely to receive maternal care at institutions with poor performance [37,38] and to give birth at hospitals that perform poorly on quality metrics compared to their white counterparts [39]. Birth location is estimated to contribute to 48% of the racial differences in severe maternal morbidity rates in New York City [37]. The differences in outcomes are likely due to variations in care delivery, such as obstetrical practices (e.g., use of oxytocin, episiotomy, anesthesia) [40], and cesarean birth rates [41].

With regard to usability, specifications for the vast majority of measures had not been updated in the year prior to our review. One in four measures had not been updated in more than 5 years. Regular updates are critical to ensure that diagnosis and procedure codes align with the most current versions of ICD, CPT, and other code sets. They are also important to ensure that clinical practices evaluated in process measures align with the most current society recommendations and clinical practice guidelines [42].

Usability is further impacted by the low number of measures that have benchmarks, are optimized to compare performance for consumers, and can be used to certify providers on safety and quality standards. The fact that less than one-fifth of measures had publicly available benchmarks is particularly concerning. Benchmarks are critical for meaningful performance evaluation, as they make an individual provider’s or facility’s performance easier to interpret by users and help organizations set goals for their quality improvement initiatives. There also remains a need for more measures that rely on electronic health records than on paper records, which can improve measure feasibility and promote the use of health information technology. Further development of EHR-based measures depends in part on the ability of clinics to transition away from paper records. Since the implementation of the Health Information Technology for Economic and Clinical Health Act in 2009 and the 21^st^ Century Cures Act in 2016, EHR adoption has occurred in nearly all non-federal acute care hospitals and nearly 80% of office-based physicians in the USA [43]. Challenges to adoption of EHR remain in smaller and rural facilities.

Stakeholder engagement in decision-making, planning, design, governance, and delivery of healthcare services is widely advocated as an important pillar in improving healthcare delivery. Globally, principles of stakeholder engagement are entrenched in Alma-Ata’s call for full participation of individuals and communities in healthcare [44]. Furthermore, in the USA, the Patient-Centered Outcomes Research Institute (PCORI) prioritizes patient and other stakeholder input in the research process [16,45]. Engaging patients can inform provider education and enhance service delivery and policies [15]. However, patients report viewing their involvement as tokenistic when the decision-making process is advanced or decisions have already been made [46].

Quality measure development has historically been the responsibility of health professionals and their respective professional organizations, as patients have been viewed as having limited expertise in quality of care and subject matter expertise [47,48]. To the best of our knowledge, our study is the first to engage patient stakeholders in the process of systematically assessing maternal and infant health quality measures. Our findings highlight the importance of engaging patients in both the prioritization of areas for maternal health measure development and the selection of measures for comparing performance of clinics and providers for consumers [49].

This study had several strengths that enhanced the rigor of our methods and credibility of our findings. We employed a team-based coding approach to improve the reliability of coding decisions and iteratively generate new versions of the codebook, which provided an audit trail for documenting changes. Our interdisciplinary team of researchers, clinicians, and citizen scientists ensured that coding and interpretation of measures accounted for the perspectives of diverse stakeholders. The study was conducted across teams with over 20 years of experience evaluating quality of care in the Florida and Texas Medicaid programs and benefited from the team’s expertise in quality-of-care measurement and maternal health.

This study also had several limitations. Measures reviewed in this study were identified in 2020. At the time of publication, four postpartum neonatal measures have since lost NQF endorsement [IDs 41, 45, 58, 71]. Other measures that would have met the study inclusion criteria have also been developed since our review. These include one new postpartum measure developed by the University of California, San Francisco (SINC-Based Contraceptive Care) and one new intrapartum Joint Commission measure (ePC-07: Severe Obstetric Complications). Furthermore, in March 2023, CMS discontinued funding for the NQF-QPS, which was a major source of measures for this study. NQF no longer endorses or maintains quality performance measures and cannot be used to replicate measure identification findings. The new endorsing entity for CMS, Partnership for Quality Measurement (https://p4qm.org/), has developed a searchable repository measure database that includes most NQF-indexed measures.

Due to low rates of valid responses among clinician collaborators, this review did not include the “payment” policy lever, which assesses whether a measure can be used to reward or incentivize providers. This study would have benefited from including a health economist with healthcare operations expertise on the team. Lastly, coding of the “mortality” element for maternal measures relied on the list of top causes of maternal mortality in Florida. Therefore, our study’s findings on mortality-related measures may not reflect the full scope of issues facing mothers nationally.

## Conclusion

This structured review study found there is a sufficient number of publicly available maternal and infant healthcare quality measures to address the immediate needs of reducing mortality, improving safety, and comprehensively assessing care in the postpartum period. However, we also identified several deficiencies in the feasibility and usability of these measures. Furthermore, most measures of clinical practice are designed to evaluate single, isolated interventions. Developing a comprehensive program for quality improvement requires selecting a set of measures to cover the broader spectrum of maternal and infant healthcare. Our findings on individual measures can be used to help providers, clinics, and facilities select measure sets for their own programs that are tailored to the specific needs of populations they serve.

Moving forward, findings from this study can (1) inform the specification of a comprehensive, updated system for maternal and infant quality-of-care evaluation and (2) facilitate the development of new quality-of-care measures that address underrepresented maternal and infant health issues.

## Supporting information

Theis et al. supplementary material Theis et al. supplementary material

## Supplementary material

The supplementary material for this article can be found at https://doi.org/10.1017/cts.2024.681.
