# Supplementary material for: Meaningfulness, feasibility, and usability of quality-of-care measures for maternal and infant health: A structured mixed-methods review
Source: J Clin Transl Sci. 2024 Dec 18;9(1):e17. doi: 10.1017/cts.2024.681 (PMC11795855; doi:10.1017/cts.2024.681)
Supplement: Theis et al. supplementary material [file S2059866124006812sup001.docx]

**Meaningfulness, Feasibility, and Usability of Quality-of-Care Measures for Maternal and Infant Health: A Structured Mixed-Methods Review**

Ryan P. Theis, PhD^1,2*^; Rahma S. Mkuu, PhD^1^; Hannah Marmol^1^; Lauren Silva, MD^3^; Callie Reeder, MD^3^; Jessica Bahorski, PhD, ARPN^4^; Erica Smith, MD^3^; John C. Smulian, MD^3,5^; Tony S. Wen, MD^3,5^; Amanda Redinger^2^; Tabresha Blake^2^; Elizabeth A. Shenkman, PhD^1,2^; Dominick J. Lemas, PhD^1,2,3,5^

^1^ Department of Health Outcomes and Biomedical Informatics, College of Medicine, University of Florida, Gainesville, FL

^2^ Clinical and Translational Science Institute Learning Health System Program, College of Medicine, University of Florida

^3^ Department of Obstetrics & Gynecology, College of Medicine, University of Florida​

^4^ College of Nursing, Florida State University, Tallahassee, FL

^5^ Center for Research in Perinatal Outcomes, University of Florida

*Corresponding author to whom correspondence should be addressed.

Author to whom correspondence should be addressed:

Ryan Theis, Ph.D.

Assistant Professor

Department of Health Outcomes and Biomedical Informatics

University of Florida College of Medicine

1889 Museum Rd., Suite 7000, Office 7110

Gainesville, FL 32611

Ph: 352-294-5973

Email: rtheis@ufl.edu

# REVIEW METHODOLOGY

## Measures identification

Measures relevant to maternal and infant quality of care were identified through resources available through the National Quality Forum (NQF), the Agency for Healthcare Research and Quality (AHRQ), the Centers for Medicare and Medicaid Services (CMS), and the National Committee for Quality Assurance (NCQA). Measure identification took place between June and September 2020.

All team members offered input on search terms and search criteria to identify measures. At each resource, we searched for measures using the following terms: “ante-partum”, “birth”, “delivery”, “deliveries”, “infant”, “maternal”, “maternity”, “mother”, “neonatal”, “neonate”, “newborn”, “perinatal”, “post-partum”, “pregnancy”, “pregnant”, “prenatal”, “pre-partum”. For hyphenated terms, both hyphenated (e.g., “post-partum”) and non-hyphenated (e.g., “postpartum”) versions were included in the search.

**NQF.** Measures indexed by the National Quality Forum were identified using the NQF Quality Positioning System (QPS) (<https://www.qualityforum.org/Qps/>). The following steps were followed:

1. Each search term was combined with “Target Population” parameters of “Children” or “Women”, and all measures meeting these criteria were collected.
2. To identify relevant measures for which a target population is not indicated in the QPS database, the same queries were made without “Target Population” parameters set; any previously unidentified measures with the search term(s) in the name of the measure were then added to the list.
3. Measures in the following NQF Measure Portfolios were collected, and any that were not previously identified were added to the list: HRSA Maternal and Child Health Bureau Measures; OB/GYN; Partnership for Patients: Maternity Care/Obstetrical Adverse Events; Perinatal and Women’s Health.
4. Any identified measures that had more than one entry in the QPS database (e.g., for separate data collection methods) were de-duplicated by selecting the measure with the most recent “Updated” date.

**AHRQ.** Measures indexed by the AHRQ were identified using the AHRQ All-Payer Claims Database (APCD) Measure Inventory (<https://www.ahrq.gov/data/apcd/measureinventory.html>) and the AHRQ-CMS Pediatric Quality Measures Program (PQMP) (<https://www.ahrq.gov/pqmp/measures/index.html>).

- The AHRQ APCD Measure Inventory includes 300 measures that can be calculated from APCDs, which are large state databases that include claims, eligibility, and provider data collected from private and public payers. The measure inventory dataset was exported into Excel format, and all measures with “Childbirth or Reproductive Health” listed in the “Condition or Focus” field were collected (n = 19). Additional measures with any parameter listed in the “Condition or Focus” field and any of the study search terms in the measure name were then collected.
- The AHRQ-CMS PQMP indexes over 150 measures relevant to children’s healthcare quality developed by PQMP Centers of Excellence (COE) grantees. The full list of PQMP measures was copied into Excel format, and all measures with the following values in the “Measure Domain” field were collected: “Availability of Services: High-Risk Obstetrics” (n = 7), “Management of Acute Conditions: Temperatures of Low Birthweight Neonates” (n = 4), “Management of Acute Conditions: Neonatal Intensive Care” (n = 3), and “Perinatal Care” (n = 7). Additional measures with any parameter listed in the “Measure Domain” field and any of the study search terms in the measure name were then collected.

**CMS.** Measures indexed by CMS were identified using the CMS Measures Inventory Tool (MIT) (<https://cmit.cms.gov/CMIT_public/ListMeasures>). All measures for which the “Conditions” field was populated with “Pregnancy” were collected (n = 16). Next, the CMS Core Set of Maternal and Perinatal Health Measures for Medicaid and CHIP (Maternity Core Set) for 2020 were collected. Then, the CMS-MIT was again queried with no restrictions, and measures containing any of the search terms in the measure names not previously identified were collected.

**NCQA**. Measures were identified from the NCQA Health Effectiveness Data and Information Set (HEDIS 2020)( <https://www.ncqa.org/hedis/measures/>). All measures with measure names including any of the search terms were collected (n = 4).

**Exclusions.** The search identified 153 unique measures. Measure names and descriptions (as listed in the first resource in which they appeared) were reviewed for face validity and excluded according to population- and condition-based criteria. Any measure that did not have at least one of the study search terms in the measure name was subject to the following exclusions:

1. **Population.** Measures were excluded if they did not specifically address one of the two following populations: (1) women of childbearing age, defined as age 15 through 49 years; or (2) infants and toddlers, up through age 3 years.^[[1]](#endnote-1)^ An exception to this exclusion was made for measures that directly address perinatal care for women who fall outside the specified age range or for which no age specifications are provided.
2. **Conditions – maternal.** For women of childbearing age, measures were excluded if they did not address one or more of the leading causes of maternal morbidity or mortality, and/or one or more common maternal complications and conditions during pregnancy, as listed below.
   1. *Causes of maternal morbidity or mortality –* hemorrhage, infection or sepsis, amniotic fluid embolism, thrombotic pulmonary/other embolism, hypertensive disorders of pregnancy, anesthesia complications, cerebrovascular accidents, cardiovascular disease.^[[2]](#endnote-2)^
   2. *Common maternal complications* – anemia, urinary tract infections, depression, hypertension, preeclampsia, gestational/pre-gestational diabetes, obesity and weight gain, infections, HIV, viral hepatitis, sexually transmitted infections, tuberculosis, hyperemesis gravidarum, preterm labor, pregnancy loss/miscarriage, and stillbirth.^[[3]](#endnote-3),^^[[4]](#endnote-4)^
3. **Conditions –** **infant.** For infants and toddlers in the indicated age range, measures were excluded if they did not address one or more of the most common causes of infant mortality, and/or conditions related to the healthy development of infants and toddlers (and for which infants and toddlers are commonly screened), as listed below.
   1. *Causes of infant mortality* – birth defects, preterm birth or low birth weight, accidents.^[[5]](#endnote-5)^
   2. *Conditions related to the healthy development of infants and toddlers* – autism spectrum disorders; birth defects; cancer; cerebral palsy; chickenpox; common cold; conjunctivitis; developmental disabilities; diabetes; diphtheria; down syndrome; ear infection; fetal alcohol spectrum disorders; influenza; foodborne diseases; group B strep; hand, foot, and mouth disease; hearing loss; hepatitis A; hepatitis B; Hib disease; intellectual disability; jaundice; parasitic diseases; pertussis; recreational water illness; rotavirus; SIDS; sinus infection; spina bifida; and vision impairment.^[[6]](#endnote-6)^

When reviewing measure descriptions for potential exclusion based on conditions, measures that address preventive care or primary care generally (e.g., well-care visits, access to PCPs) were considered to address one or more of the listed conditions and were not excluded on this basis. Certain measures were excluded from the list for other reasons:

- Medical Home System Survey (MHSS) and Wrong-Patient Retract-and-Reorder (Wrong Patient-RAR) were excluded because the measure denominators are not patient-level.
- C-Section Rates (NH CHIS) and Obstetrics: Risk-Adjusted Rate of Urgent Readmission for the Obstetric Patient Group (NQMC:010031) were excluded because no description or specification of the measure was publicly available to determine measure eligibility.

Furthermore, with input from subject-matter experts in obstetrics and gynecology, we opted to exclude measures that are solely based on cost and utilization and not otherwise based on a standard of care, as these measures do not provide an indication of maternal or infant health care quality. Information on measure type (e.g., cost, utilization, structure, process, or outcome) was collected from documentation produced by measure developers.

After exclusions, this process identified 88 measures relevant to maternal or infant health, and which were included in the study for further review.

## Measures review

All measures were reviewed following a framework to collect information on measure specifications and properties, meaningfulness, feasibility, and usability. The initial version of the framework included 60 fields. We used a concurrent, independent review method following a team-based coding methodology and framework analysis methods that are suited for structured qualitative data reduction.^[[7]](#endnote-7),^^[[8]](#endnote-8)^ Measure elements were abstracted using an Excel template that included fields in five domains of measure elements:

1. Measure identifiers and specifications included information on the measure steward, date of last update, and the measure numerator and denominator specifications. Additionally, each measure was classified as relevant to the structure, process, or outcomes of care.^[[9]](#endnote-9)^ Each measure was also assigned a focus (women, neonates, infants, and toddlers) and a phase (preconception, prenatal, intrapartum, postpartum, and interpregnancy).
2. Evidence and support included information on NQF endorsement and ratings and recommendations on clinical practices from the American Academy of Pediatrics (AAP), American College of Obstetricians and Gynecologists (ACOG), Centers for Disease Control and Prevention (CDC), Society for Maternal-Fetal Medicine (SMFM), and U.S. Preventive Services Task Force (USPSTF).
3. Meaningfulness was assessed following the AHRQ National Quality Strategy (NQS) priorities of safety, engagement, coordination, mortality, community, and affordability.^[[10]](#endnote-10)^ Additionally, clinical investigators with subject matter expertise (LS, CR, JB, ES, TSW) and citizen scientist investigators from the UF CTSI Citizen Scientist Program (AR, TB) rated each measure on its “importance to maternal health” on a 5-point Likert scale.
4. Feasibility was assessed using AHRQ guidance for evaluating measure feasibility, including consistent measure construction and assessment, feasibility of calculating (based on the measure data source and availability of measure diagnosis and procedure codes), and addressing confidentiality concerns.^[[11]](#endnote-11)^
5. Usability was coded in two sub-domains – practice usability (the extent to which providers, clinics, and health systems can incorporate the measure into practice) and policy usability (the extent to which policymakers can use measure findings to inform policy).
   1. Practice usability was assessed using AHRQ guidance for evaluating measure usability, including measure presentation, history of use, and compelling content for stakeholder decision-making,^[[12]](#endnote-12)^ as well as the availability of measure benchmarks and the level(s) at which the measure is aligned (e.g., provider, facility, system).
   2. Policy usability included the NQS “levers” of feedback, public reporting, learning, certification, consumer incentives, payment, health information technology, innovation, and workforce development.^[[13]](#endnote-13)^ The public reporting element, which specifies whether a measure can be used to inform patient decision-making by comparing performance of providers and clinics, was coded by citizen scientist investigators (AR, TB).

Measure coding was deductive and followed an iterative cycle for codebook development. The coding team was comprised of two independent reviewers who abstracted information on measure specifications and determined whether measures met the criteria for meaningfulness, feasibility, and usability (RPT, RSM), using a measures framework table developed in MS Excel. Differences in coding were reconciled by consensus between the coders and during team meetings, which included the study lead (DJL), clinical investigators (JCS, TSW), and citizen scientist investigators (AR, TB). A coding lead (RPT) compiled abstracted measure information, calculated inter-coder reliability (ICR) statistics, and updated the final measures framework table during the review period.

Coding for this study was deductive and proceeded according to a three-step cycle:

1. **Coding and reliability assessment.** First, two coders were each assigned a subset of the same measures (~one-fifth of the full set of identified measures) to independently review and abstract using the Excel template and codebook. After coding this subset of measures, the coding lead collected both completed templates and compiled the information to enable side-by-side comparisons. For each coded measure, the coding lead calculated: (a) observed agreement on Yes/No fields in the *meaningfulness*, *feasibility*, and *usability* domains; (b) the total observed agreement across these fields; and (c) Cohen’s Kappa statistic, which represents an overall measure of ICR, accounting for the possibility of agreement occurring by chance. An average Cohen’s Kappa statistic was also calculated across all measures reviewed.
2. **Coding resolution.** The coding team (both independent reviewers and a third team member) then met to discuss and resolve any discrepancies in coding. In cases where the independent reviewers maintained disagreement on a particular coding discrepancy, the third team member made the final determination for coding. After all coding was discussed, the coding lead produced a clean version of the final framework table with rows for all measures and all data populated.
3. **Codebook refinement**. Based on decisions made during the coding meeting, the team then refined the codebook. Changes to the codebook included updates to element definitions, review criteria, or the ranges of values. In some cases, these refinements involved expanding review criteria to ensure better consistency in coding for certain elements. Decisions were also made for consolidating measure elements that were shown to be redundant, or for removing measure elements for which objective review criteria could not be established.

The coding team followed the same three-step cycle for the next one-fifth of measures, generating a new version of the codebook as needed based on team discussion. In cases where subsequent changes to the codebook necessitated revisions to coding done during the preceding cycle, reviewers back-coded the elements in question in the final framework table. The coding team followed this process for each increment of one-fifth of the total pool of measures until all measures were reviewed. Overall, the process involved five coding cycles and meetings, and six versions of the codebook.

Measures were selected for coding cycles following a purposeful sampling approach, with measures grouped according to measure type (structure, process, outcome), maternal-infant health phase (preconception, prenatal, intrapartum, postpartum, and interpregnancy), and measure focus (woman, neonates, infants, and toddlers).

Initial intercoder reliability ranged broadly across measures, from κ = 0.06 (Cycle 1, Measure #35) to κ = 1.00 (Cycle 3, Measures #33, #34, and #40). The overall mean was κ = 0.57. The mean increased from κ = 0.37 in Cycle 1 to κ = 0.61 in Cycle 5. All disagreements for a measure were reconciled before adding the measure to the final framework table.

# MEASURES INCLUDED IN REVIEW

| **ID** | **Measure Title** | **Measure type** | **Measure focus** | **Measure phase** | **Measure Steward** |
| --- | --- | --- | --- | --- | --- |
| 1.0 | Appropriate DVT prophylaxis in women undergoing cesarean delivery | Process | Women | Intrapartum | HCA |
| 2.0 | Appropriate Prophylactic Antibiotic Received Within One Hour Prior to Surgical Incision – Cesarean section. | Process | Women | Intrapartum | MGH |
| 3.0 | Audiological Evaluation no later than 3 months of age, aka Audiological Diagnosis No Later Than 3 Months of Age | Process | Neonatal, Infant | Postpartum | CDC |
| 4.0 | Behavioral Health Risk Assessment for Pregnant Women | Process | Women | Prenatal | PMCOE |
| 5.0 | Birth dose of hepatitis B vaccine and hepatitis B immune globulin for newborns of hepatitis B surface antigen (HBsAg) positive mothers | Process | Neonatal | Postpartum | CDPH |
| 6.0 | Birth Trauma – Injury to Neonate (PSI 17) | Outcome | Neonatal | Intrapartum | AHRQ |
| 7.0 | BMI Assessment and Recommended Weight Gain | Process | Women | Prenatal | PMCOE |
| 8.0 | CAPQuaM PQMP HROB I: High risk deliveries at facilities with 24/7 in-house physician capable of safely managing labor and delivery, and performing a cesarean section, including an emergent cesarean section | Structure | Women | Intrapartum | CAPQuaM |
| 9.0 | CAPQuaM PQMP HROB II: High risk deliveries at facilities with 24/7 in-house physician coverage dedicated to the obstetrical service by an anesthesiologist who is qualified to provide obstetrical anesthesia | Structure | Women | Intrapartum | CAPQuaM |
| 10.0 | CAPQuaM PQMP HROB III: High risk deliveries at facilities with 24/7 in-house blood banking/transfusion services available | Structure | Women | Intrapartum | CAPQuaM |
| 11.0 | CAPQuaM PQMP HROB IV: High risk deliveries at facilities with level 3 or higher NICU services on campus | Structure | Women | Intrapartum | CAPQuaM |
| 12.0 | CAPQuaM PQMP HROB V: Availability of outpatient maternal fetal medicine and specialty care for women with high risk pregnancies | Structure | Women | Prenatal | CAPQuaM |
| 13.0 | CAPQuaM PQMP HROB VI: Availability of multidisciplinary outpatient care for women with high risk pregnancies | Structure | Women | Prenatal | CAPQuaM |
| 14.0 | CAPQuaM PQMP HROB VII: Assessing the availability of the preconception component of High Risk Obstetrical Services by Estimating the Use of Teratogenic Medications Before and During Pregnancy | Structure | Women | Preconception | CAPQuaM |
| 15.0 | CAPQuaM PQMP PERINATAL I: Timely temperatures for all low birthweight neonates | Process | Neonatal | Postpartum | CAPQuaM |
| 16.0 | CAPQuaM PQMP PERINATAL II: Timely temperatures upon arrival in Level 2 or higher nurseries for low birthweight neonates | Process | Neonatal | Postpartum | CAPQuaM |
| 17.0 | CAPQuaM PQMP PERINATAL III: Distribution of temperatures for low birthweight neonates admitted to Level 2 or higher nurseries in the first 24 hours of life | Outcome | Neonatal | Postpartum | CAPQuaM |
| 18.0 | CAPQuaM PQMP PERINATAL IV: Thermal condition of low birth weight neonates admitted to Level 2 or higher nurseries in the first 24 hours of life | Outcome | Neonatal | Postpartum | CAPQuaM |
| 19.0 | Cesarean Delivery for Nulliparous (NTSV) Women (Appropriate Use) | Outcome | Women | Intrapartum | PMCOE |
| 20.0 | Childhood Immunization Status (CIS) | Process | Toddler | Interpregnancy | NCQA |
| 21.0 | Chlamydia Screening and Follow Up | Process | Women | Preconception | NCQA |
| 22.0 | Chlamydia Screening in Women (CHL) | Process | Women | Preconception | NCQA |
| 23.0 | Continuity of Insurance - Duration of Newborns' First Observed Enrollment | Structure | Infant, Toddler | Postpartum, Interpregnancy | CHOP |
| 24.0 | Contraceptive Care - Access to LARC | Process | Women | Preconception | OPA |
| 25.0 | Contraceptive Care – Most & Moderately Effective Methods | Process | Women | Preconception | OPA |
| 26.0 | Contraceptive Care - Postpartum - Access to LARC | Process | Women | Postpartum | OPA |
| 27.0 | Counseling for Women of Childbearing Potential with Epilepsy | Process | Women | Preconception | AAN |
| 29.0 | Death Rate in Low-Mortality Diagnosis Related Groups (PSI02) | Outcome | Women | All | AHRQ |
| 30.0 | Diabetes and Pregnancy: Avoidance of Oral Hypoglycemic Agents | Process | Women | Prenatal | Resolution Health, Inc. |
| 31.0 | Episiotomy (Overuse) | Outcome | Women | Intrapartum | PMCOE |
| 32.0 | Establishment of Gestational Age | Process | Women | Prenatal | PMCOE |
| 33.0 | First NICU Temperature < 36 degrees Centigrade | Outcome | Neonatal | Postpartum | VON |
| 34.0 | First temperature measured within one hour of admission to the NICU. | Process | Neonatal | Postpartum | VON |
| 35.0 | Frequency of Ongoing Prenatal Care (FPC) | Process | Women | Prenatal | NCQA |
| 36.0 | Hearing screening prior to hospital discharge | Process | Neonatal | Postpartum | CDC |
| 37.0 | Hepatitis B Vaccine Coverage Among All Live Newborn Infants Prior to Hospital or Birthing Facility Discharge | Process | Neonatal | Postpartum | CDC |
| 38.0 | Incidence of Episiotomy | Outcome | Women | Intrapartum | CCHS |
| 39.0 | Intrapartum Antibiotic Prophylaxis for Group B Streptococcus (GBS) | Process | Women | Intrapartum | MGH |
| 40.0 | Late sepsis or meningitis in neonates (risk-adjusted) | Outcome | Neonatal | Postpartum | VON |
| 41.0 | Late sepsis or meningitis in Very Low Birth Weight (VLBW) neonates (risk-adjusted) | Outcome | Neonatal | Postpartum | VON |
| 42.0 | Low Birth Weight Rate (PQI 9) | Outcome | Neonatal | Postpartum | AHRQ |
| 43.0 | Maternal Depression Screening | Process | Women | Postpartum | NCQA |
| 44.0 | Maternity Care: Elective Delivery or Early Induction Without Medical Indication at < 39 Weeks (Overuse) | Outcome | Women | Intrapartum | CMS |
| 45.0 | Neonatal Blood Stream Infection Rate (NQI 03) | Outcome | Neonatal | Postpartum | AHRQ |
| 46.0 | Neonatal Immunization | Process | Neonatal | Postpartum | CHCA |
| 47.0 | Neonatal Intensive Care All-Condition Readmissions with Gestational Age Reported | Outcome | Neonatal | Postpartum | CHOP |
| 48.0 | Neonatal Intensive Care All-Condition Readmissions without Gestational Age | Outcome | Neonatal | Postpartum | CHOP |
| 49.0 | Neonatal Intensive Care Outcomes | Outcome | Neonatal | Postpartum | CHOP |
| 50.0 | Neonate immunization administration | Process | Neonatal | Postpartum | CHCA |
| 51.0 | Newborn Hearing Screening | Process | Infant | Postpartum | NCQA |
| 52.0 | Outpatient hearing screening of infants who did not complete screening before hospital discharge (EHDI-1c) | Process | Neonatal, Infant | Postpartum | CDC |
| 53.0 | PC-01 Elective Delivery | Process | Women | Intrapartum | TJC |
| 54.0 | PC-02 Cesarean Birth | Process | Women | Intrapartum | TJC |
| 55.0 | PC-03 Antenatal Steroids | Process | Women | Prenatal | TJC |
| 56.0 | PC-04 Health Care-Associated Bloodstream Infections in Newborns | Outcome | Neonatal | Postpartum | TJC |
| 57.0 | PC-05 Exclusive Breast Milk Feeding, including subset PC-05a Considering Mother's Choice | Process | Women,  Neonatal | Postpartum | TJC |
| 58.0 | Percentage of low birthweight births, aka Live Births Weighing Less than 2,500 Grams | Outcome | Neonatal | Postpartum | CDC |
| 59.0 | Post-Partum Follow-Up and Care Coordination | Process | Women | Postpartum | PMCOE |
| 60.0 | Pregnancy test for female abdominal pain patients. | Process | Women | Preconception | ACEP |
| 61.0 | Pregnant women that had HBsAg testing | Process | Women | Prenatal | Ingenix |
| 62.0 | Pregnant women that had HIV testing | Process | Women | Prenatal | Ingenix |
| 63.0 | Pregnant women that had syphilis screening | Process | Women | Prenatal | Ingenix |
| 64.1 | Prenatal & Postpartum Care (PPC) - Prenatal | Process | Women | Prenatal | NCQA |
| 64.2 | Prenatal & Postpartum Care (PPC) - Postpartum | Process | Women | Postpartum | NCQA |
| 65.0 | Prenatal Anti-D Immune Globulin | Process | Women | Prenatal | PCPI |
| 66.0 | Prenatal Blood Group Antibody Testing | Process | Women | Prenatal | PCPI |
| 67.0 | Prenatal Blood Groups (ABO), D (Rh) Type | Process | Women | Prenatal | PCPI |
| 68.0 | Prenatal Care Screening | Process | Women | Prenatal | PMCOE |
| 69.0 | Prenatal Screening for Human Immunodeficiency Virus (HIV) | Process | Women | Prenatal | PCPI |
| 70.0 | Promoting Healthy Development Survey (PHDS) | Process | Infant, Toddler | Interpregnancy | CAHMI |
| 71.0 | Proportion of infants 22 to 29 weeks gestation screened for retinopathy of prematurity. | Process | Neonatal | Postpartum | VON |
| 72.0 | Proportion of infants 22 to 29 weeks gestation treated with surfactant who are treated within 2 hours of birth. | Process | Neonatal | Postpartum | VON |
| 73.0 | Proportion of infants covered by Newborn Bloodspot Screening (NBS) | Process | Neonatal | Postpartum | HRSA |
| 74.0 | Rh immunoglobulin (Rhogam) for Rh negative pregnant women at risk of fetal blood exposure | Process | Women | Prenatal | ACEP |
| 75.0 | Severity-Standardized ALOS - Deliveries | Outcome | Women | Intrapartum | Leapfrog Group |
| 76.0 | Signed Part C Individual Family Service Plan (IFSP) before 6 months of age | Process | Neonatal, Infant | Postpartum | CDC |
| 77.0 | Standardized mortality ratio for neonates undergoing non-cardiac surgery | Outcome | Neonatal | Postpartum | CPSQR |
| 78.0 | Sudden Infant Death Syndrome Counseling | Process | Women,  Neonatal, Infant | Postpartum | NCQA |
| 79.0 | Transfusion Reaction Count (PSI 16) | Outcome | Women | Prenatal, Intrapartum, Postpartum | AHRQ |
| 80.0 | Ultrasound determination of pregnancy location for pregnant patients with abdominal pain | Process | Women | Prenatal | ACEP |
| 81.0 | Under 1500g infant Not Delivered at Appropriate Level of Care | Outcome | Neonatal | Intrapartum | CMQCC |
| 82.0 | Unexpected Complications in Term Newborns, aka Heathy Term Newborn | Outcome | Neonatal | Postpartum | CMQCC |
| 83.0 | Ventilator-associated pneumonia for ICU and high-risk nursery (HRN) patients | Outcome | Neonatal | Postpartum | CDC |
| 84.0 | Well-Child Visits in the First 15 Months of Life (W15-CH) | Process | Toddler | Interpregnancy | NCQA |
| 85.0 | Contraceptive Care – Postpartum - Most & Moderately Effective Methods | Process | Women | Postpartum | OPA |
| 86.0 | Prenatal Immunization Status | Process | Women | Prenatal | NCQA |
| 87.0 | Prenatal Depression Screening and Follow-up | Process | Women | Prenatal | NCQA |
| 88.0 | Postpartum Depression Screening and Follow-up | Process | Women | Postpartum | NCQA |

# ENDNOTES

1. The three-year cutoff was selected for infants and toddlers based on CDC guidance for parents (<https://www.cdc.gov/parents/infants/index.html>). [↑](#endnote-ref-1)
2. <https://www.cdc.gov/reproductivehealth/maternal-mortality/pregnancy-mortality-surveillance-system.htm>. [↑](#endnote-ref-2)
3. <https://www.cdc.gov/reproductivehealth/maternalinfanthealth/pregnancy-complications.html>. [↑](#endnote-ref-3)
4. <https://www.nichd.nih.gov/health/topics/pregnancy/conditioninfo/complications>. [↑](#endnote-ref-4)
5. <https://www.cdc.gov/nchs/data/nvsr/nvsr68/nvsr68_10-508.pdf>. [↑](#endnote-ref-5)
6. <https://www.cdc.gov/parents/infants/diseases_conditions.html>. [↑](#endnote-ref-6)
7. Giesen L, Roeser A. 2020. Structuring a Team-Based Approach to Coding Qualitative Data. *Int J Qual Methods*, 19. doi:10.1177/1609406920968700. [↑](#endnote-ref-7)
8. Gale NK et al. 2013. Using the framework method for the analysis of qualitative data in multi-disciplinary health research. *BMC Medical Research Methodology*, 13: 117. [↑](#endnote-ref-8)
9. Ayanian JZ, Markel H. Donabedian’s Lasting Framework for Health Care Quality. N Engl J Med. 2016;375(3):205-207. doi:10.1056/NEJMp1605101 [↑](#endnote-ref-9)
10. AHRQ. Six Domains of Health Care Quality. Agency for Healthcare Research and Quality. Published December 2022. Accessed November 17, 2023. <https://www.ahrq.gov/talkingquality/measures/six-domains.html> [↑](#endnote-ref-10)
11. AHRQ. Guidance for Using the AHRQ Quality Indicators for Public Reporting or Payment - Appendix B: Public Reporting Evaluation Framework -- Comparison of Recommended Evaluation Criteria in Five Existing National Frameworks. Published 2004. <https://qualityindicators.ahrq.gov/Downloads/Modules/QI_reporting/Model_Report_Appendix_B.pdf> [↑](#endnote-ref-11)
12. AHRQ 2004. [↑](#endnote-ref-12)
13. AHRQ. National Quality Strategy Fact Sheet. Published 2017. <https://www.ahrq.gov/sites/default/files/wysiwyg/nqsfactsheet_2017.pdf> [↑](#endnote-ref-13)
